# The evolution of ventral intermediate nucleus targeting in MRI-guided focused ultrasound thalamotomy for essential tremor: an international multi-center evaluation

**DOI:** 10.3389/fneur.2024.1345873

**Published:** 2024-03-26

**Authors:** Ayesha Jameel, Sena Akgun, Nada Yousif, Joely Smith, Brynmor Jones, Dipankar Nandi, Peter Bain, Wladyslaw Gedroyc

**Affiliations:** ^1^Imperial College London, London, United Kingdom; ^2^Imperial College Healthcare NHS Trust, London, United Kingdom; ^3^Sapienza University of Rome, Rome, Italy; ^4^University of Hertfordshire, Hatfield, United Kingdom

**Keywords:** magnetic resonance guided focused ultrasound (MRgFUS), essential tremor (ET), movement disorders, tremor, ventral intermediate nucleus (VIM), thalamotomy, stereotactic targeting, tractography

## Abstract

**Background:**

The ventral intermediate nucleus (VIM) is the premiere target in magnetic resonance-guided focused ultrasound (MRgFUS) thalamotomy for tremor; however, there is no consensus on the optimal coordinates for ablation. This study aims to ascertain the various international VIM targeting approaches (VIM-TA) and any evolution in practice.

**Methods:**

International MRgFUS centers were invited to share VIM-TAs in 2019 and 2021. Analyses of any modification in practice and of anatomical markers and/or tractography in use were carried out. Each VIM-TA was mapped in relation to the mid-commissural point onto a 3D thalamic nucleus model created from the Schaltenbrand–Wahren atlas.

**Results:**

Of the 39 centers invited, 30 participated across the study period, providing VIM-TAs from 26 centers in 2019 and 23 in 2021. The results are reported as percentages of the number of participating centers in that year. In 2019 and 2021, respectively, 96.2% (*n* = 25) and 95.7% (*n* = 22) of centers based their targeting on anatomical landmarks rather than tractography. Increased adoption of tractography in clinical practice and/or for research was noted, changing from 34.6% to 78.3%. There was a statistically significant change in VIM-TAs in the superior-inferior plane across the study period; the percentage of VIM-TAs positioned 2 mm above the intercommissural line (ICL) increased from 16.0% in 2019 to 40.9% in 2021 (WRST, *p* < 0.05). This position is mapped at the center of VIM on the 3D thalamic model created based on the Schaltenbrand–Wahren atlas. In contrast, the VIM-TA medial-lateral and anterior-posterior positions remained stable. In 2022, 63.3% of participating centers provided the rationale for their VIM-TAs and key demographics. The centers were more likely to target 2 mm above the ICL if they had increased experience (more than 100 treatments) and/or if they were North American.

**Conclusion:**

Across the study period, FUS centers have evolved their VIM targeting superiorly to target the center of the VIM (2 mm above the ICL) and increased the adoption of tractography to aid VIM localization. This phenomenon is observed across autonomous international centers, suggesting that it is a more optimal site for FUS thalamotomy in tremors.

## Introduction

The ventral intermediate nucleus (VIM) of the thalamus is currently established as the premiere target for magnetic resonance-guided focused ultrasound (FUS) thalamotomy in essential tremor (ET). Over the past decade, the efficacy of FUS VIM ablation has been proven in multiple international studies (1–3) and recent systematic reviews, which demonstrated pooled tremor suppression of 56.7%, 62.4%, and 61.5%, respectively (4–7). Although other tremor-specific targets such as the cerebellothalamic tract (8) or even a combination of targets such as the VIM with the posterior subthalamic area (PSA) (9, 10) have been explored, the VIM alone remains the most frequently used target in FUS treatment for ET (11, 12). Several alternative targets have been considered in Parkinson's disease (PD) (13–16), but the VIM remains the target of choice for FUS treatment of tremor-dominant PD (17). The success of VIM ablation can be readily seen with its global adoption and the growth in the number of FUS centers performing thalamotomy for tremors (18).

The VIM, a motor nucleus within the lateral thalamic subgroup of nuclei, is a proven tremor-sensitive nucleus within the cerebello-thalamo-cortical network (19, 20). In FUS thalamotomy, accurate targeting of the VIM is crucial to ensure adequate tremor suppression while avoiding the erroneous ablation of adjacent structures, risking motor and sensory adverse effects. The VIM is predominantly bordered anteriorly by the ventral oralis posterior (VOP), a motor nucleus in the pallidothalamocortical pathway (21, 22), and posteriorly by the ventralis caudalis (VC), a large sensory nucleus (21, 23). The medial border of the VIM is less well defined, but on the Schaltenbrand–Wahren (S-W) atlas (21), it includes the ventro-oralis internus, the lamella medialis interpolaris, and the nuclei centrales thalami. The VIM's lateral border is the nucleus reticularis, a thin strip of tissue separating the VIM from the internal capsule (IC), which contains the pyramidal tracts. On the S-W atlas, the superior border of VIM is predominantly formed by the nucleus zentrolateralis intermedius. The fasciculus interstitio-thalamicus, the zona incerta (ZI), and the prelemniscal radiation (RAPRL) form the inferior border of the VIM and are included on the coronal plates of the S-W atlas. The ZI and RAPRL are often considered together as the PSA. Although some centers deliberately target PSA, a cautious approach should also be taken to inferior lesioning as there is a risk of chorea (9, 10). Consideration of the posterior and lateral borders is also crucial in VIM targeting to minimize the risk of ablation of key adjacent structures and associated adverse sensory and motor effects.

Unfortunately, current clinical MRI scanners at 1.5 and 3 Tesla (T) cannot delineate the VIM on conventional MRI pulse sequences. Although post-processing techniques have allowed the demarcation of thalamic nuclei subgroups on 3T MRI (20), individual nuclei cannot be determined. Therefore, VIM targeting in FUS traditionally relies on anatomical landmarks to infer the VIM position. The key structures used are demonstrated across all brain cross-sectional imaging modalities and include the third ventricle and internal capsule (IC). The cerebrospinal fluid-filled third ventricle borders the thalamus medially. The IC is a large confluence of white matter tracts that, on CT and MRI, form a distinct lateral border to the thalamus, which itself is a large confluent region of gray matter. The anterior commissure (AC) and posterior commissures (PC) are relatively thin white matter tracts that cross the cerebral hemispheres, which can be visualized on specific MRI sequences, including the Fast Gray Matter Acquisition T1 Inversion Recovery (FGATIR) sequence (24) or the Magnetization Prepared RApid Gradient Echo sequence (MP-RAGE) (25). In the midline, the AC–PC line or intercommissural line (ICL) is an imaginary line joining these two structures and is widely used as an imaging plane and as the baseline for stereotactic neurosurgery. As the inferior border of the VIM lies close to the axial plane projected at the level of the ICL, it can readily be inferred on an MRI.

The traditional approach to VIM targeting in FUS thalamotomy utilizes the ICL to set the superior-inferior (SI) position with pre-determined measurements in anterior-posterior (AP) and medial-lateral (ML) positions along this trajectory. FUS treatment allows the target to be adjusted according to patient response, in sub-millimeter increments, before a permanent ablation is performed. This technique has been well described in the literature (26) and allows the tailoring of treatments to individual neuroanatomy. The most commonly published or “traditional” VIM targeting approach (VIM-TA) utilizes the following trajectories: (AP) 25% of ICL length, anterior to PC; (ML) 14–16 mm lateral; and (SI) on the ICL plane. However, as clinical experience grows, FUS centers naturally adapt their VIM-TA. For example, our centre's approach has evolved over 7 years of practice from the described traditional VIM-TA to (AP) 3–5 mm posterior to MCP; (ML) 3–5 mm medial to IC; and (SI) 2 mm above ICL in 2023. At this site, we achieve better tremor control with minimal adverse effects and are completing treatments in fewer sonications with a shorter procedural time. This learning from experience, or “evolution”, will have occurred at every FUS center; however, there is currently no published data describing the various VIM-TAs used internationally, and an update is vital.

Diffusion tensor imaging (DTI) or “tractography” is an imaging technique that utilizes the anisotropic diffusion properties of water in the white matter tracts to create three-dimensional maps of neural pathways and provide information on directionality. As the VIM lies between several large white matter tracts, the medial lemniscus, the pyramidal tract, and the dentatorubothalamic tract, some FUS centers use tractography to infer the VIM position (27–30). Tractography promises highly individualized VIM-TA; however, it is not yet universally adopted. Of note, tractography does not directly visualize the VIM, but an ultrahigh-field strength MRI at 7T can provide enough contrast between thalamic nuclei to delineate the VIM (31). Current clinical scanners operate at 1.5T and 3T; unfortunately, 7T MRIs are not readily available in healthcare institutions, so direct VIM visualization remains within the research space. DTI and ultrahigh-field MRI offer patient-specific targeting, and future developments in these and other advanced imaging techniques may lead to higher adoption.

Early FUS publications focused on the safety and efficacy of this novel treatment for tremor, with a paucity of data on the technique itself. With safety and efficacy established (1, 2), there has been a notable trend in scientific output from FUS centers with more detailed technical methodology, including closer reporting of their approach to VIM targeting (32, 33). There has also been further enquiry into improving FUS treatments from a technical perspective (34), including specific consideration of skull factors (29, 35–37), thermal dose and lesion size (38–41), and imaging aspects (42–45). However, there has not yet been a review or consensus on the optimum location for FUS VIM ablation. To establish this, an evaluation of VIM-TAs utilized internationally and documentation of the evolution of FUS centres' practice are the natural first step. Furthermore, sharing any such analysis based on clinical experience gained with the optimal FUS technique is vital to ensure improved tremor suppression and minimization of adverse effects for ET patients treated with FUS.

## Aims

This article aims to ascertain the various international approaches to targeting the VIM (VIM-TA) in magnetic resonance-guided focused ultrasound (FUS) thalamotomy for essential tremor (ET) and consider how targeting has evolved internationally as experience develops.

## Materials and methods

Between July 2019 and July 2021, all 39 MRgFUS centers from the Insightec Limited (Haifa, Israel) international FUS tremor database were invited to participate in this study and share their VIM targeting approach (VIM-TA). Each FUS center was contacted at least three times via email. Invitations included reassurance that participating FUS centres' contributions would be acknowledged in any subsequent academic output from this study, but individual VIM-TAs would remain anonymous. Where possible, the system operator (neurosurgeon and/or neuroradiologist) was contacted directly. At many centers, correspondence was first conducted through clinical or research administrators before reaching the appropriate clinician. The best efforts were made to ensure the system operator provided the VIM-TA where initial contact was via a third party. Written informed consent from the participants was not required to participate in this study in accordance with national legislation and institutional requirements. Ethical review and approval were not required for the study in accordance with the local legislation and institutional requirements.

Participants were invited to share their VIM-TA for 2019 and 2021 with open correspondence rather than a rigid questionnaire to encourage the sharing of information and discussion.

1. Please describe your approach to VIM targeting.
  ° *If anatomical targeting is used, what landmarks and distances (in mm) are used?*
  ° *If there is an alternative targeting method, please describe*.
2. Do you use tractography?

Where the VIM-TA was anatomical, coordinates were calculated with respect to the mid-commissural point (MCP) to allow 3D modeling and mapping graphically. Coordinates were determined in three planes, namely, anterior-posterior (AP), medial-lateral (ML), and superior-inferior (SI), with the MCP considered coordinate 0 in all three axes. Positive numbers were assigned to anterior, lateral, and superior movements. Negative numbers were assigned for medial, posterior, and inferior movements. This method was chosen to accommodate various ICL lengths. Where centers provided a range (mm) for a specific plane, the mid-point was taken.

Where tractography-based targeting was reported, technical details of the methodology were requested. Where tractography was used to complement anatomical-based targeting, the primary anatomical-targeting method was mapped.

VIM-TAs were mapped graphically and on a 3D model created from the S-W atlas (21). The model was created from Brain LXXVIII, Axial Plates 53–55. Images were stacked in MATLAB (R2021a, MathWorks Inc.) to obtain uniform resolution in three dimensions, and using the 3D slicer (v4.11.2021022), key thalamic nuclei were segmented, including VIM, VOP, and VC, and the model surfaces were smoothed. The model ICL length was scaled to a modern average of 27.8 mm (based on the last 30 MRgFUS patients at our center), and the model coordinates were scaled accordingly prior to mapping.

The results were first analyzed with regard to VIM-TA, whether anatomical and/or using tractography. Further analysis considered VIM-TA coordinates in relation to MCP, both graphically and within key nuclei on the 3D model. Finally, any change, trend, or evolution in VIM-TA across the study period was determined.

In 2022, all participating centers were invited to share further details on their experience with FUS thalamotomy, the rationale for their VIM-TA, and any change in practice. Further analysis of the centres' years of experience, number of treatments performed, and geography was conducted to determine whether any correlation to the trends in VIM-TA is ascertained.

## Results

Across the study period, a total of 30 participants from the database of 39 centers participated (Table 1), with a response rate of 76.9%. Complete VIM-TA was reported by 26 centers for 2019 (Appendix 1), 23 centers for 2021 (Appendix 2), and 20 centers provided data for both years. For each year cohort (2019 and 2021), the results are provided as percentages of the number of participating centers in that year. The majority of the centers replied with direct answers describing VIM-TA, but some provided presentation slides, unpublished data summaries, and published papers. Where appropriate, replies were clarified with responders directly before converting VIM-TAs to coordinates for mapping. Two centers were excluded from the coordinate analysis as complete VIM-TAs could not be determined.

**Table 1:** List of participating FUS centers in alphabetical order (please note this does not correlate to center number).

**Country**	**Participating MRgFUS centers (in Alphabetical Order)**
Italy	Azienda Ospedaliera Universitaria Integrata Verona
USA	Brigham and Women's Hospital
Taiwan	Chang Bing Show Chwan Memorial Hospital
Taiwan	CMUH (China Medical University Hospital)
Italy	Fondazione IRCCS Istituto Neurologico Carlo Besta Milano
Spain	HM CINAC, Hospital HM Puerta del Sur
Japan	Hokuto Hospital
UK	Imperial College Healthcare NHS Trust
USA	Mayo Clinic
Canada	Montreal Neurological Institute and Hospital
USA	NYU Langone Health
USA	Ohio State University
USA	Penn Medicine
Israel	Rambam Medical Center
Japan	Sadamoto Hospital
Japan	Saito Yukokai Hospital
Israel	Sheba Medical Center
USA	Sperling Medical Center
Australia	St. Vincent's Hospital, Sydney
USA	Stanford University Hospital
USA	Swedish Hospital
USA	University of Maryland
USA	University of Utah
Italy	University Degli Studi Di Palermo
Switzerland	University Hospital Zurich
Germany	University of Bonn
Canada	University of Calgary
Italy	University of L'Aquila
Canada	University of Toronto
South Korea	Yonsei University College of Medicine

**Figure 1:**
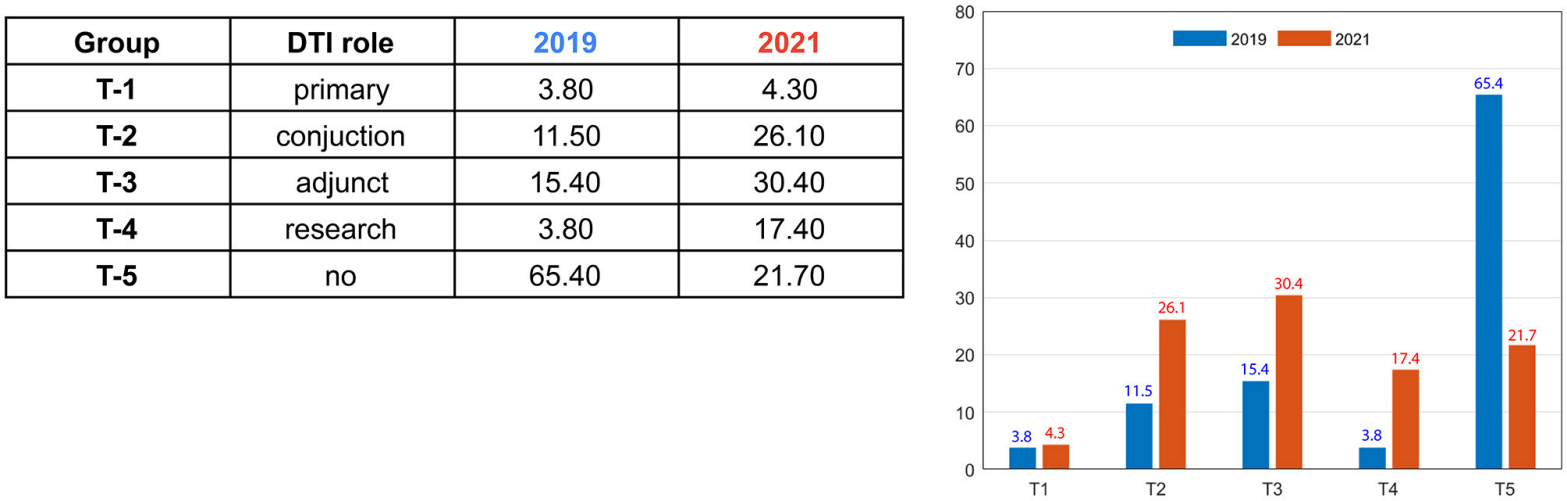
Use of tractography in FUS thalamotomy for tremor.

The vast majority of FUS centers used anatomical VIM-TAs: 96.2% in 2019 (*n* = 25) and 95.7% in 2021 (*n* = 22), with only one center using primarily tractography-based VIM-TA. However, across the study period, more centers incorporated tractography in conjunction with or as an adjunct to their anatomical targeting (Figure 1). In 2019, only 30.7% were utilizing tractography in their clinical practice, and in 2021, this doubled to 60.8% (total groups T1–T3). Participating centers using tractography only for research increased more than four-fold across the study period, from only 3.8% in 2019 to 17.4% in 2021 (Group T-5). Furthermore, the percentage of centers not using tractography in any role decreased from 65.4% to 21.7%. The one center with a tractography-based VIM-TA shared its published papers, which included a well-described methodology (27, 28). As the S-W atlas does not delineate individual white matter tracts, this centre's VIM-TA was not mapped onto the 3D model.

**Figure 2:**
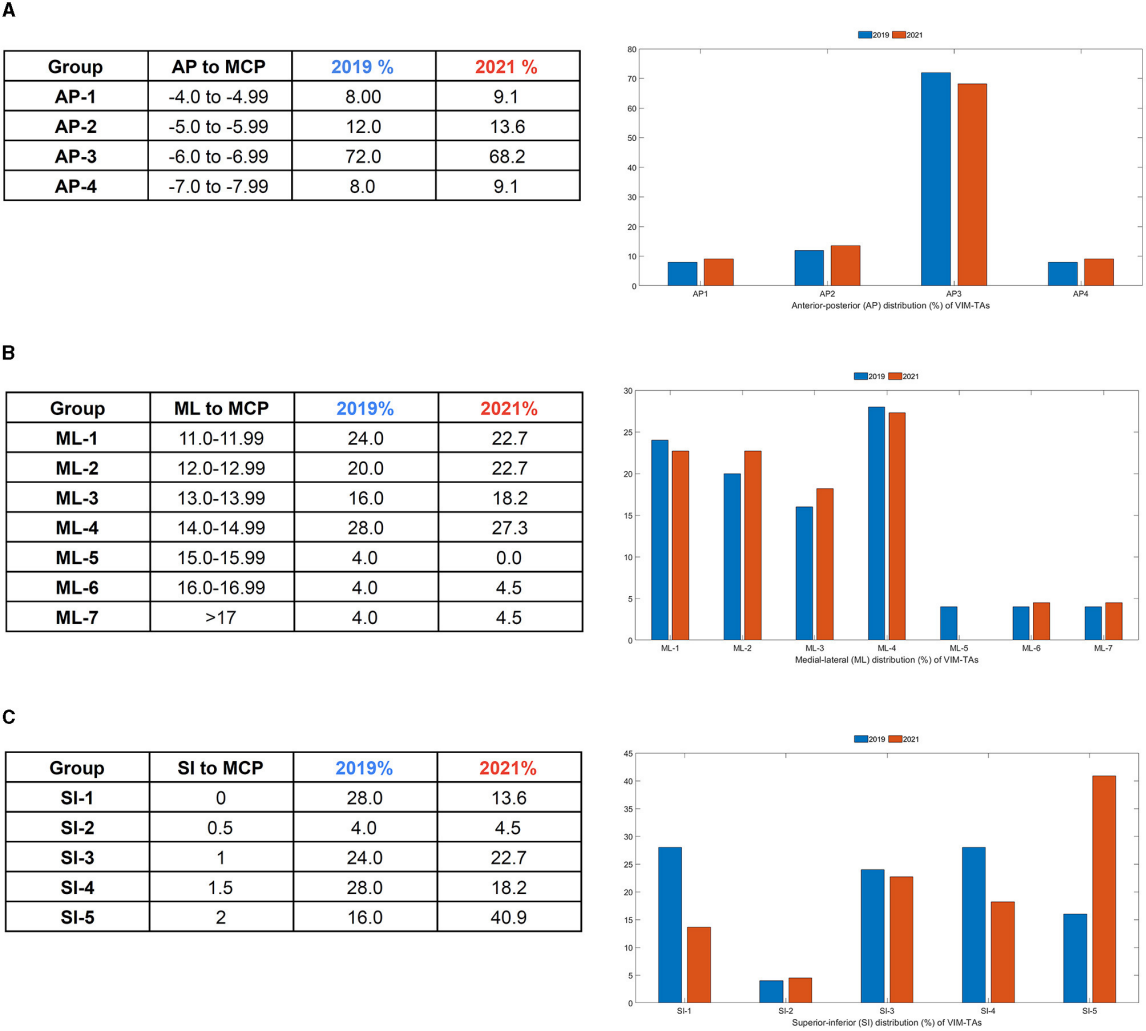
Distribution of VIM-TA coordinates in relation to the Midcommissural point (MCP) as a **(A)** Anterior-Posterior (AP) % distribution, **(B)** Medial-Lateral (ML) % distribution, and **(C)** Superior-Inferior (SI) % distribution.

**Figure 3:**
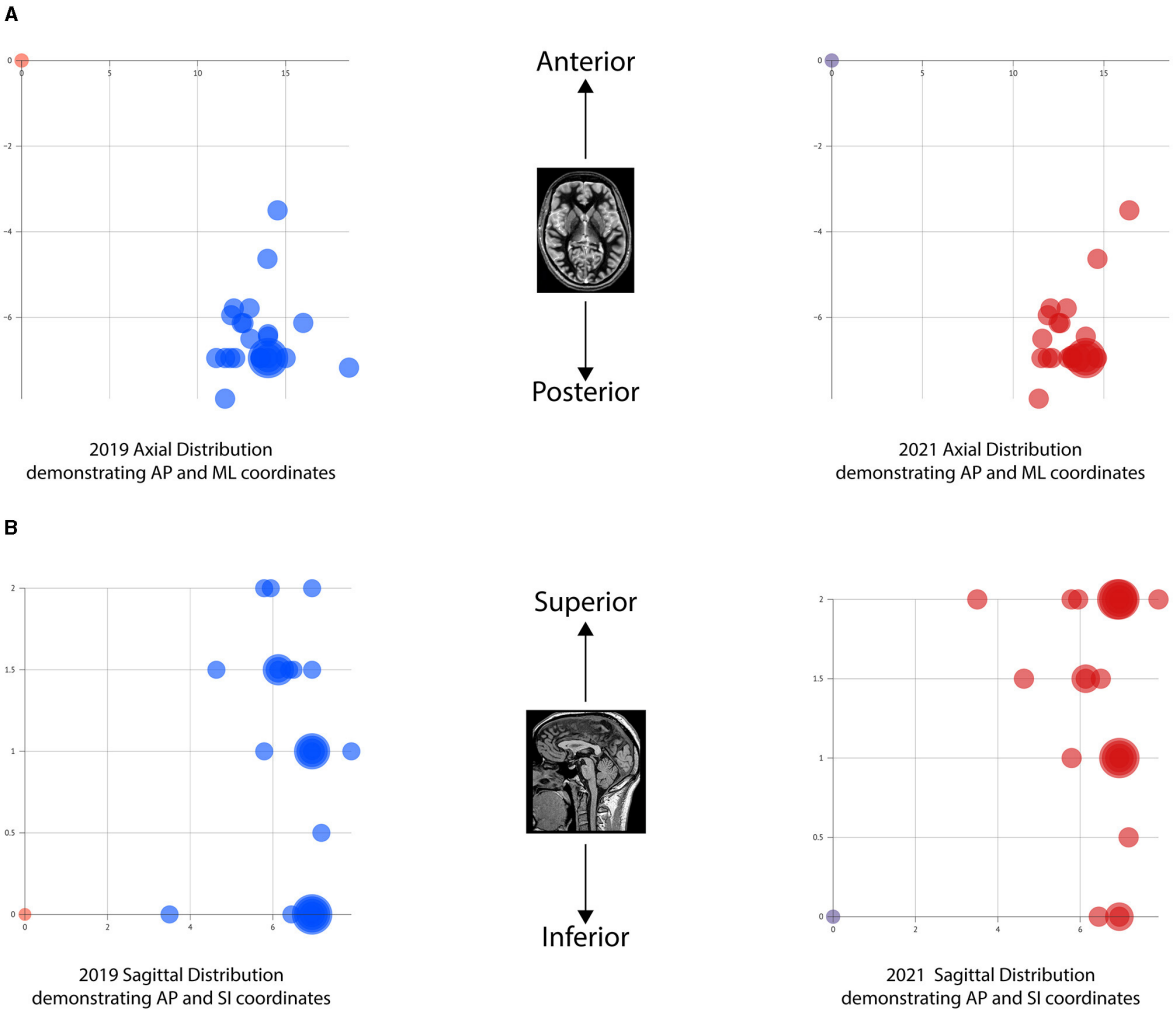
Graphical mapping of the VIM-TAs. **(A)** Axial graphs of VIM-TA coordinates in relation to midcommissural point, demonstrating anterior-posterior (AP) and medial-lateral (ML). **(B)** Sagittal graphs of VIM-TA coordinates in relation to midcommissural point, demonstrating anterior-posterior (AP) and superior-lateral (SI).

**Figure 4:**
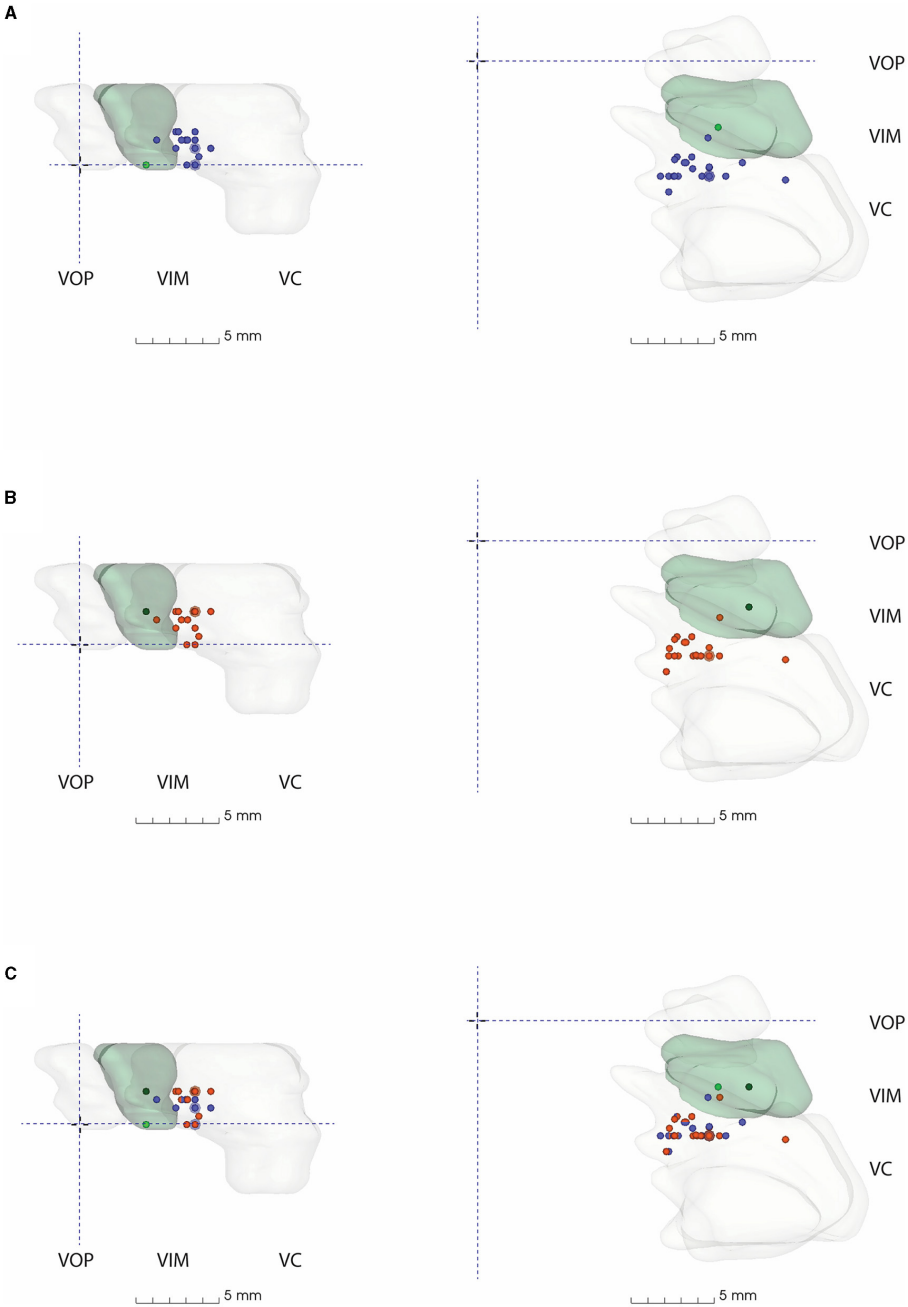
3D model of thalamic nuclei with participating centre's VIM-TAs mapped **(A)** 2019, **(B)** 2021, and **(C)** 2019 + 2021. MCP, Mid-commissural point; VOP, Ventral oralis posterior; VIM, Ventral Intermediate Nucleus; VC, Ventral Caudalis.

All anatomical VIM-TAs were calculated in relation to the MCP (considering ICL length) prior to analysis. The distribution of anatomical VIM-TA coordinates in the AP, ML, and SI planes was tabulated and graphically demonstrated (Figures 2A–C). All VIM-TAs were mapped onto the axial and sagittal graphs (Figures 3A, B) and the 3D thalamic nucleic model (Figures 4A–C); full coordinates are listed in Appendices 1, 2.

### Anterior-posterior plane

For both 2019 and 2021, the majority of centers targeted the VIM from −6 to −6.9 mm posterior to the MCP (Group AP-3), accounting for 72% of centers in 2019 (*n* = 18) and 68.2% of centers in 2021 (*n* = 15) (Figure 2A). Of note, the AP position was relatively fixed over the study period, with no statistically significant change noted on a Wilcoxon Signed Rank Test (WRST) (*p* = 0.865), although some centers diverged slightly along the ML and SI planes (Figures 3A, B).

### Medial-lateral plane

The majority of centers targeted VIM between 11.0 and 14.9 mm lateral to the MCP (Groups ML-1 to ML-4), accounting for 88% of centers in 2019 (*n* = 22) and 90.9% of centers in 2021 (*n* = 20) (Figure 2B). For both years, 14.0–14.9 mm lateral to the MCP (Group ML-4) was the most common position (Figure 3B). The distribution of ML coordinates was stable across the study period, with no statistically significant change in WRST (*p* = 0.779).

### Superior-inferior plane

Across the study period, there was a statistically significant superior migration in VIM targeting in the SI plane (Figures 2B, C). In 2019, there were only 16% of centers (*n* = 4) targeting 2 mm above the ICL; this increased to 40.9% of centers (*n* = 9) in 2021 (group SI-5) [WRST, *p* = 0.046 (*p* < 0.05)]. Conversely, 28% of centers (*n* = 7) targeted the ICL in 2019, and this decreased to 13.6% of centers (*n* = 3) in 2021. Of note, those who were already targeting at 2 mm did not move, suggesting that this location is viewed as the optimal tremor lesioning site. One center moved inferiorly (from 1.5 mm to ICL).

### 3D model

All FUS centers utilizing an anatomical VIM-TA were mapped onto a 3D model created from the S-W Brain LXXVIII (as described in methodology), whose VIM measures approximately 5 mm (AP) × 8 mm (ML) × 5.5 mm (SI) (17). The model demonstrates the non-uniform shape of the VIM, with smooth tapering inferiorly in both the AP and ML dimensions. The model includes the anterior VOP and the larger posterior VC nuclei. Of note, the size and shape of the thalamic nuclei modeled are specific to the S-W Brain LXXVIII. The VIM-TAs (Appendices 1, 2) were mapped onto the model in relation to MCP for 2019, 2021, and both years combined (Figure 4, Appendix 3).

On Brain LXXVIII, the most common AP position (Group AP-3), between −6 and −6.9 mm posterior to MCP, lies within the anterior VC. There are several centers that target more anteriorly to this position at the VIM/VC junction, but only two centers where model coordinates lie within VIM itself on Brain LXXVIII (AP-1). No VIM-TAs were modeled within the VOP. In the ML plane, the most common position (ML-4) lies within the medial aspect of the key nuclei; there were no laterally placed VIM-TAs to suggest encroachment on IC.

The superior trend for targeting in the SI plane, from the ICL to 2 mm above the ICL, is well demonstrated in the model (Figures 4A, B) by the number of centers moving from the inferior border of VIM (Group SI-1) to the middle of VIM (Group SI-5) across the study period. Given the inferior tapering of the VIM in the AP and ML planes, targeting at 2 mm above the ICL is shown to be more centrally placed within the VIM. The dark green dots represent our centre's VIM-TA (Imperial), which evolved from the ICL in 2019 to 2 mm above the ICL in 2021.

## Discussion

Across the study period, the VIM-TAs evolved more superiorly to 2 mm above the ICL. This movement occurred independently across autonomous international FUS centers, with a combined experience exceeding 1,800 treatments. This change developed as experience accrued, presumably reflecting the view that this superior target provides better tremor suppression and/or minimizes adverse effects. The S-W 3D model also supports this concept, demonstrating the natural inferior tapering of VIM in the AP and ML planes (Figure 4). As VIM-TAs at 2 mm above the ICL lie more centrally within the VIM, sonications here will ablate more VIM tissue than at the ICL. Interestingly, centers with the least experience were most likely to move their VIM-TA across the study period, suggesting that the evolution of VIM-TAs tends to occur within the first 100 FUS treatments (Table 2B).

**Table 2:** FUS Center experience.

**(A) FUS Center experience (to date 1st January 2022) in relation to SI co-ordinates**.						
**No. procedures**	**Total centers**	**On ICL**	**0.5 mm above**	**1 mm above**	**1.5 mm above**	**2 mm above**
>100	7	28.8%	0%	0%	14.3%	57.1%
50–100	7	0%	0%	42.9%	14.3%	42.9%
< 50	3	0%	33.3%	0	33.3%	33.3%
**(B) FUS Center experience (to date 1st January 2022) and movement over the study period**.		
**No. procedures**	**Total centers**	**Movement**
>100	7	0%
50–100	7	28.7%
< 50	3	66.7%

**Figure 5:**
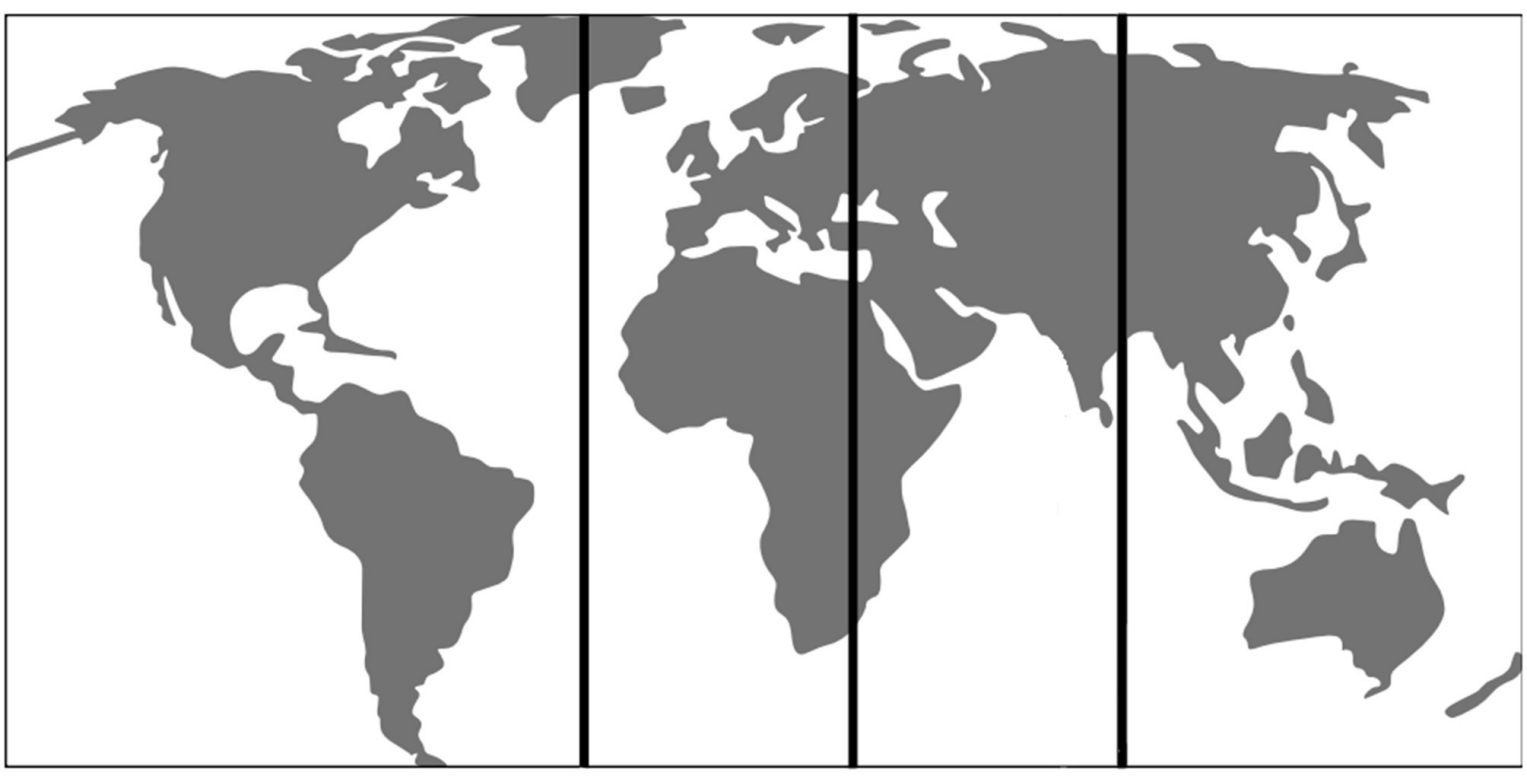
World Map demonstrating the 4 regions used for geographical analysis of VIM-TAs. R1 = Region 1 which includes North American centers, R2 = Region 2 which includes European centers, R3 = Region 3 which includes West Asian centers, R4 = Region 4 which includes East Asian and Australian centers.

In 2022, 63.3% of participating centers (*n* = 19) provided the rationale for their VIM-TAs and key demographics, including the number of treatments performed (to date, 1 January 2022); a detailed analysis of this data is provided in Appendix 5. In summary, in 2021, the centers were more likely to target 2 mm above the ICL if they had increased experience (more than 100 treatments) (Table 2A) and/or if they were North American (rather than European or Asian) (Table 3, Figure 5). The reported rationales for VIM-TAs (Table 4, Appendix 4) included improved tremor suppression and a reduction in adverse effects or safety. Some centers reported that their VIM-TAs were influenced by their prior experience with deep brain stimulation (DBS) or gamma knife (GK). Others discussed the size, shape, and risk of cranial-caudal extension of the FUS sonication spot. Many centers reported that moving superiorly allowed them to perform a second, more inferior lesion in the same FUS procedure (which is also the practice at our centre at Imperial). The solitary center that moved inferiorly from 2019 to 2021 reported in their rationale high sensory adverse effects and a possible second ablation below ICL (Table 4, Appendices 4, 5). Interestingly, of the three centers targeting ICL who provided their rationale, all reported performing a second target (in the same FUS procedure) if tremor suppression was inadequate, either superior or inferior to ICL (Table 4B). Although these findings are of interest, not all participating centers provided a rationale for their VIM-TA, reducing the significance of these summations, and thus, clinical conclusions can only be drawn to a limited extent.

**Table 3:** FUS center regional demographics.

**(A) 2019 Regional VIM-TA SI co-ordinate distribution (mm above ICL)**.						
**Center geography**	**Number of centers**	**0.0 mm**	**0.5 mm**	**1 mm above**	**1.5 mm above**	**2 mm above**
R-1	10	30%	0%	40%	10%	20%
R-2	8	25%	0%	25%	25%	25%
R-3	1	100%	0%	0%	0%	0%
R-4	6	16.7%	16.7%	0%	66.7%	0%
**(B) 2021 Regional VIM-TA SI co-ordinate distribution (mm above ICL)**.						
**Center geography**	**Number of centers**	**0.0 mm**	**0.5 mm**	**1 mm**	**1.5 mm**	**2 mm**
R-1	9	0%	0%	33.3%	0%	66.7%
R-2	7	28.6%	0%	28.6%	14.3%	28.6%
R-3	0	0%	0%	0%	0%	0%
R-4	6	16.7%	16.7%	0%	50%	16.7%

**Table 4:** FUS centers rationale for 2021 VIM-TA.

**(A) Table of FUS centers with rationale**.						
**Center Number**		**SI coordinate 2021**		**Rationale**		
1		2 mm		1 + 2 + 5		
4		2 mm		2		
10		2 mm		1 + 2 + 5		
12		2 mm		2		
13		2 mm		4		
14		2 mm		2 + 5		
15		2 mm		1 + 5		
26		2 mm		1 + 2		
28		2 mm		1 + 2 + 3 + 5		
27		1.5 mm ^*^moved to 2mm in 2022 for safety		2		
19		1.5 mm		4 + 5 (avoid the lesion extending below AC-PC.)		
21		1.5 mm		1 + 3		
5		1 mm		5 (target higher (2 mm) for 2nd side)		
22		1 mm		1		
23		1 mm		2 + 4 + 5		
20		0.5 mm		1		
8		0 mm		1 + 2 + 3 + 5		
9		0 mm		1 + 3		
17		0 mm		1 + 2 + 3 + 4		
Rationale categories: 1, improved tremor suppression. 2, reduce adverse effects/safety. 3, to allow a second target. 4, based on previous neurosurgical experience (DBS/GK). 5, other.						
**(B) FUS centers rationale with VIM-TA 2021 SI co-ordinate distribution (mm above ICL)**.						
**2021 SI coordinates**	**Total center number**	**Rationale category**				
		**1**	**2**	**3**	**4**	**5**
0 mm	3	100%	66.7%	100%	33.3%	33.3%
0.5 mm	1	100%	0%	0%	0.%	0%
1 mm	3	33.3%	33.3%	0%	33.3%	66.7%
1.5 mm	3	33.3%	33.3%	33.3%	33.3%	33.3%
2 mm	9	55.6%	77.8%	11.1%	11.1%	55.6%

1, improved tremor suppression.

2, reduce adverse effects/safety.

3, to allow a second target.

4, based on previous neurosurgical experience (DBS/GK).

5, other.

### Tractography

Although the vast majority of the centers used anatomical VIM-TAs (96.2% in 2019; 95.7% in 2021), there was an increase in the adoption of tractography in clinical practice as an adjunct or in conjunction with anatomical targeting from 26.9% in 2019 to 56.5% in 2021 (Figure 1). Many centers reported specific challenges in incorporating tractography into their practice, including being time-consuming with mixed reliability, requiring specific software and expertise, and having difficulty integrating it with the current FUS systems. Some centers only utilized tractography retrospectively to review challenging cases; however, many centers reported its potential benefits and/or a desire to use tractography when it became more reliable and easier to incorporate. Future developments in DTI and/or other imaging techniques may change the preferred choice between anatomical and tractography-based VIM-TA. Future advanced imaging may allow precise target planning, for example, by combining DTI with high-field strength MRI 7T, which allows direct visualization of individual thalamic nuclei anatomy.

### Targeting vs. clinical outcomes (tremor suppression/adverse effects)

There is an ongoing debate on the classification and functional anatomy of thalamic nuclei (46), and this study reveals several interesting findings, specifically the 3D modeling of S-W Brain LXXVIII (Figure 4). Of note, it was beyond the scope of this study to collate technical data on energy delivered, ablative spot size, and clinical outcomes. Thus, analyses of final ablation locations and clinical outcomes, including adverse effects, were not performed.

1. Targeting in the traditional AP location (25% of ICL, anterior to PC) is demonstrated on the 3D model to lie within the anterior VC, which is understood to be a sensory nucleus, yet multiple studies targeting at this location report good tremor suppression (4, 5). This finding can be explained by the size of the sonication spot created, typically approximately 3.9 mm (38), which would include the adjacent motor VIM and cause the reported tremor suppression. Interestingly, multiple studies that describe targeting based on the traditional method have reported high rates of paraesthesia; meta-analyses by Mohammed et al. and Giordano et al. observed 15.3% and 36.7% paraesthesia, respectively, which correlates with the 3D model findings (4, 5) demonstrating VIM-TAs in the anterior VC and at the VIM/VC junction. Given the ongoing discussion on the optimal AP position, the 3D model supports the suggestion that targeting anterior to the traditional location would avoid paraesthesia while achieving good tremor suppression (9).
2. Targeting in the traditional ML location (14–16 mm lateral to the ICL) has a comfortable margin from the IC. Therefore, sonication spot size should be considered when reviewing the reported 10.5% and 34.4% ataxia/gait disturbance reported in the aforementioned meta-analyses (4, 5, 9). Of note, the more modern “2–4 mm from IC” approach lies in a similar position to traditional VIM-TAs on 3D modeling. Although the 3D model did not include changes secondary to age-related brain parenchymal atrophy (which can be observed in older ET patients who undergo FUS treatment), this was considered by many centers that reported their VIM-TAs with allowances for an enlarged third ventricle, providing coordinates from its lateral border rather than the MCP itself.
3. Targeting in the traditional SI location, on the ICL plane, is demonstrated on the 3D model to lie at the inferior border of VIM. The more modern VIM-TA, 2 mm above ICL, is demonstrated at the midpoint of VIM in the SI axis. Outcomes from this study show the international evolution of VIM-TAs to this location, where there is more VIM tissue. Interestingly, depending on sonication spot size, traditional VIM ablation at ICL may extend superiorly to mid-VIM or inferiorly to the posterior subthalamic area, which includes the zona incerta, a known tremor sensitive tissue targeted in DBS and FUS (9, 47).

### Understanding the FUS lesion morphology

The results of this study should be considered in the historical context of stereotactic neurosurgical treatments for tremors. At centers with experience in radiofrequency ablation (RFA) or DBS, one would typically place the tip of the probe or electrode at the ICL to target the VIM. In RFA, the lesion created could be extended superiorly by withdrawing the probe, thereby ablating the more central portions of VIM. With DBS, this position sited the most effective electrode contacts at the center of VIM. However, in FUS, the sonication creates a sphere that expands concentrically around the target. Haray et al. have neatly demonstrated the average FUS lesion volume on immediate post-procedure MRI to be ~3.9 mm (range 1.5–6.3 mm) (38), and Gallay et al. have described their FUS targeting accuracy between 0.29 and 0.44 mm in the three dimensions of space (48). Thus, if the FUS target coordinates are placed at the ICL, the average FUS lesion would extend beyond the inferior margins of the VIM into the PSA. As demonstrated by the 3D model in this study, moving the VIM-TA to 2 mm above the ICL corresponds to a more centrally placed lesion within the VIM and, equally importantly, creates a lesion that is almost completely confined to the borders of the VIM in the superior-inferior plane. Similarly, consideration of the AP and ML dimensions of VIM and appropriate placement of the target in these planes would ensure the lesion remains within all the borders of VIM. It is important to note that ensuring a controlled, uniform expansion of the sonication spot and the ablation confined to the VIM is crucial to reducing adverse effects in FUS tremor treatments. Thus, as well as locating the best targeting coordinates, further research on optimizing sonication parameters, controlling the accumulated thermal dose, and sonication spot size and shape is required before FUS thalamotomy can be truly optimized.

### Limitations

#### COVID-19

There are several limitations to this study. First, the COVID-19 pandemic interrupted normal medical practice, including FUS treatments, across the world (49, 50), disrupting the experience and, therefore, the evolution of VIM-TAs. As global experience develops, it would be interesting to ascertain any further trends in VIM-TAs.

#### The Schaltenbrand-Wahren atlas 3D model

The accuracy of the 3D thalamic nucleus model relies on the accuracy of Brain LXXVIII from the S-W atlas (17, 34). It is important to consider that any model created from one person's brain cannot be representative of all demographics. Less information is provided about LXXVIII beyond its own demographics of a 40-year-old woman. Of note, its AC-PC length is 23 mm, which is short compared to modern brains (35); thus, the model was scaled accordingly, as described in the methodology. There are a number of brain dissections in the S-W atlas, and the VIM itself was delineated into two further dissections, one conducted in the sagittal plane and one in the coronal plane. However, as other key structures required for VIM-TA mapping were not delineated in those dissections (either VOP or MCP), the axially dissected Brain LXXVIII was chosen for the 3D model. Previous studies have demonstrated the variability in VIM size and shape within the different dissections of the S-W atlas (36), further suggesting that a variation in individual neuroanatomy should be considered in modeling.

For the macroscopic dissection of axial brain LXXVIII, the authors of the S-W model performed the dissection in Reid's plane, which differs from the AC-PC plane used for the atlas's individual plate dissections and that used in modern MRI (51). For this study, to account for various ICL lengths, all VIM-TAs were mapped in relation to MCP in the AC-PC plane. However, given the central location of the thalamus, any discrepancy between macroscopic Reid's plane and microscopic dissections in the AC-PC plane is minimal and unlikely to affect the model or VIM-TA mapping.

#### Debate on thalamic nucleic classification

There is a historical lack of consensus on thalamic nuclei classification with implications for the nomenclature of tremor targets in FUS and stereotactic neurosurgery. Although modern neurosurgery favors the Hassler classification (based on the S-W atlas), there remains considerable debate, as described by Mai et al. (46). Future studies could explore VIM-TAs mapped on other established classification systems, such as Morel's (46, 52, 53), or on individualized patient imaging, as 7T MRI allows direct VIM visualization (31). Following this, highly individualized functional thalamic neuroanatomy maps could be modeled, which when correlated with several key technical factors (including initial VIM-TA position, final VIM ablation position, sonication spot size, and clinical outcomes) would be of great value in identifying the optimal coordinates for FUS tremor treatment in ET. Recent studies have performed retrospective analyses of FUS-treated VIM positions with interesting results (54, 55); however, further research is required, including prospective studies and analyses that consider individual 3D thalamic neuroanatomy alongside tractography to better understand the optimal VIM-TA.

## Conclusion

This study demonstrates that anatomical-based targeting of VIM is the most widely utilized methodology internationally for FUS thalamotomy despite recent advances in tractography. Over the study period, there was a statistically significant superior movement to target the VIM 2 mm above the intercommissural line. This superior evolution of VIM targeting has occurred independently across autonomous international centers, suggesting that it is an optimized site for FUS thalamotomy in the treatment of tremors.

## Data availability statement

The original contributions presented in the study are included in the article/Supplementary material, further inquiries can be directed to the corresponding author.

## Author contributions

AJ: Conceptualization, Data curation, Formal analysis, Investigation, Methodology, Project administration, Resources, Supervision, Validation, Visualization, Writing—original draft, Writing—review & editing. SA: Data curation, Formal analysis, Investigation, Methodology, Visualization, Writing—review & editing. NY: Conceptualization, Data curation, Formal analysis, Methodology, Software, Validation, Visualization, Writing—review & editing. JS: Conceptualization, Methodology, Software, Visualization, Writing—review & editing, Formal analysis. BJ: Supervision, Methodology, Validation, Writing—review & editing. DN: Resources, Supervision, Writing—review & editing, Formal analysis, Methodology. PB: Resources, Supervision, Writing—review & editing, Formal Analysis, Methodology, Validation. WG: Conceptualization, Investigation, Methodology, Project administration, Resources, Supervision, Validation, Writing—review & editing, Data curation, Formal analysis.
