# Supplementary material for: The evolution of ventral intermediate nucleus targeting in MRI-guided focused ultrasound thalamotomy for essential tremor: an international multi-center evaluation
Source: Front Neurol. 2024 Mar 26;15:1345873. doi: 10.3389/fneur.2024.1345873 (PMC11002122; doi:10.3389/fneur.2024.1345873)
Supplement: Supplementary file 1 [file Data_Sheet_1.docx]

*The evolution of Ventral Intermedius Nucleus (VIM) targeting in MRI guided Focused Ultrasound (MRgFUS) thalamotomy for Essential Tremor - an international multi-centre review*

# **Appendices List**

Appendix 1:  VIM-TAs for 2019

Appendix 2:  VIM-TAs for 2021

Appendix 3:  3D model coronal views

Appendix 4: FUS Centre rationale for VIM-TA

Appendix 5:  Detailed Discussion

# **Appendix 1: 2019 VIM-TAs**

1a:  Scaled 2019 VIM-TAs for 27.8mm ICL (modern brain size) for mapping in 3D thalamic model

| **CENTRE NUMBER** | **AP** | **ML** | **SI** | **TRACTOGRAPHY** |
| --- | --- | --- | --- | --- |
| **1** | -6.95 | 11.90 | 2 | N |
| **2** | -6.95 | 11.85 | 1.5 | N |
| **3** | -6.95 | 14 | 0 | Y-A |
| **4** | -6.95 | 14 | 0 | Y- C |
| **5** | -6.95 | 11.56 | 1 | N |
| **6** | -6.95 | 11.058 | 1 | N |
| **7** | -6.95 | 13.57 | 0 | N |
| **8** | -6.95 | 12.13 | 0 | N |
| **9** | -6.45 | 14 | 0 | N |
| **10** | -5.95 | 11.91 | 2 | N |
| **11** | -6.1333333 | 16 | 1.5 | N |
| **12** | -5.791666667 | 12.95 | 2 | N |
| **13** | -7.9 | 11.56 | 1 | N |
| **14** | -6.95 | 14 | 1 | N |
| **15** | NR | NR | NR | NR |
| **16** | IC | IC | IC | Y-A |
| **17** | -6.394 | 14 | 1.5 | N |
| **18** | T | T | T | Y-P |
| **19** | -6.13333333 | 12.60 | 1.5 | Y-A |
| **20** | -7.175 | 18.6 | 0.5 | N |
| **21** | -6.13333333 | 12.5 | 1.5 | N |
| **22** | -6.95 | 14 | 1 | Y-A |
| **23** | -5.791666667 | 12.06 | 1 | N |
| **24** | -6.95 | 15 | 2 | Y-A |
| **25** | -6.95 | 13.57 | 0 | N |
| **26** | IC | IC | 0 | N |
| **27** | -4.6333333 | 13.93 | 1.5 | Y |
| **28** | -4.0 | 14.54 | 0 | N |
| **29** | -6.5 | 13 | 1.5 | N |
| **30** | IC | IC | IC | IC |

*NR= no reply, IC= incomplete VIM-TA, T- tractography based VIM-TA, N= No tractography, Y-A= Yes, tractography as an adjunct to anatomical targeting, Y- C = Yes, tractography in conjunction with anatomical targeting; Y-P = Yes, tractography as primary VIM-TA.*

1b: Scaled 2019 centre VIM-TA’s calculated to ICL 23.0mm per Schaltenbrand -Wahren Brain LXXVIII

| **CENTRE NUMBER** | **AP** | **ML** | **SI** | **TRACTOGRAPHY** |
| --- | --- | --- | --- | --- |
| **1** | -5.75 | 11.75 | 2 | N |
| **2** | -5.75 | 11.75 | 1.5 | N |
| **3** | -5.75 | 14 | 0 | Y-A |
| **4** | -5.75 | 14 | 0 | Y- C |
| **5** | -5.75 | 11.38 | 1 | N |
| **6** | -5.75 | 10.88 | 1 | N |
| **7** | -5.75 | 10.43 | 0 | N |
| **8** | -5.75 | 11.85 | 0 | N |
| **9** | -5.25 | 14 | 0 | N |
| **10** | -4.75 | 11.75 | 2 | N |
| **11** | -5.33 | 16 | 1.5 | N |
| **12** | -4.79 | 12.88 | 2 | N |
| **13** | -5.5 | 11.38 | 1 | N |
| **14** | -5.75 | 14 | 1 | N |
| **15** | NR | NR | NR | NR |
| **16** | IC | IC | IC | Y-A |
| **17** | -5.29 | 14 | 1.5 | N |
| **18** | T | T | T | Y-P |
| **19** | -5.375 | 12.5 | 1.5 | Y-A |
| **20** | -5.375 | 10.5 | 0.5 | N |
| **21** | -5.33 | 12.5 | 1.5 | N |
| **22** | -5.75 | 14 | 1 | Y-A |
| **23** | -4.79 | 11.88 | 1 | N |
| **24** | -5.75 | 15 | 2 | Y-A |
| **25** | -5.75 | 13.43 | 0 | N |
| **26** | IC | IC | 0 | N |
| **27** | -3.83 | 14 | 1.5 | Y |
| **28** | -4.0 | 10.93 | 0 | N |
| **29** | -6.5 | 13 | 1.5 | N |
| **30** | IC | IC | IC | IC |

*NR= no reply, IC= incomplete VIM-TA, T- tractography based VIM-TA, N= No tractography, Y-A= Yes, tractography as an adjunct to anatomical targeting, Y- C = Yes, tractography in conjunction with anatomical targeting; Y-P = Yes, tractography as primary VIM-TA.*

# Appendix 2: 2021 VIM-TAs

2a: Scaled 2021 VIM-TAs for 27.8mm ICL (modern brain size) for mapping in 3D thalamic model

| **CENTRE NUMBER** | **AP** | **ML** | **SI** | **TRACTOGRAPHY** |
| --- | --- | --- | --- | --- |
| **1** | -6.95 | 11.91 | 2 | Y-A |
| **2** | NR | NR | NR | NR |
| **3** | -6.95 | 13.50 | 2 | Y-A |
| **4** | -6.95 | 14 | 2 | Y-C |
| **5** | -6.95 | 11.56 | 1 | Y-A |
| **6** | NR | NR | NR | NR |
| **7** | -6.95 | 13.28 | 1 | Y-A |
| **8** | -6.95 | 12.13 | 0 | N-RESEARCH |
| **9** | -6.45 | 14 | 0 | N |
| **10** | -5.95 | 11.91 | 2 | N |
| **11** | NR | NR | NR | NR |
| **12** | -5.79 | 12.95 | 2 | Y-C |
| **13** | -7.90 | 11.41 | 2 | N |
| **14** | -6.95 | 14 | 2 | Y-A |
| **15** | -6.90 | 13.20 | 2 | Y-C |
| **16** | -6.95 | 13.03 | 1 | N |
| **17** | -6.95 | 14.62 | 0 | N |
| **18** | T | T | T | Y-P |
| **19** | -6.13 | 12.60 | 1.5 | Y-A |
| **20** | -7.18 | 18.6 | 0.5 | N |
| **21** | -6.13 | 12.5 | 1.5 | Y-C |
| **22** | -6.95 | 14 | 1 | Y-C |
| **23** | -5.79 | 12.06 | 1 | Y-A |
| **24** | NR | NR | NR | NR |
| **25** | NR | NR | NR | NR |
| **26** | IC | IC | 2 | Y-C |
| **27** | -4.63 | 14.64 | 1.5 | Y |
| **28** | -4.0 | 16.4 | 2 | N |
| **29** | -6.5 | 11.60 | 1.5 | N-RESEARCH |
| **30** | IC | IC | IC | IC |

*NR= no reply, IC= incomplete VIM-TA, T- tractography based VIM-TA, N= No tractography, Y-A= Yes, tractography as an adjunct to anatomical targeting, Y- C = Yes, tractography in conjunction with anatomical targeting; Y-P = Yes, tractography as primary VIM-TA.*

2b: Scaled 2021 centre VIM-TA’s calculated to ICL 23.0mm per Schaltenbrand -Wahren Brain LXXVIII

| **CENTRE NUMBER** | **AP** | **ML** | **SI** | **TRACTOGRAPHY** |
| --- | --- | --- | --- | --- |
| **1** | -5.75 | 11.75 | 2 | Y-A |
| **2** | NR | NR | NR | NR |
| **3** | -5.75 | 13.5 | 2 | Y-A |
| **4** | -5.75 | 14 | 2 | Y-C |
| **5** | -5.75 | 11.38 | 1 | Y-A |
| **6** | NR | NR | NR | NR |
| **7** | -5.75 | 13.19 | 1 | Y-A |
| **8** | -5.75 | 11.85 | 0 | N-RESEARCH |
| **9** | -5.25 | 14 | 0 | N |
| **10** | -4.75 | 11.75 | 2 | N |
| **11** | NR | NR | NR | NR |
| **12** | -4.79 | 12.88 | 2 | Y-C |
| **13** | -5.5 | 11.25 | 2 | N |
| **14** | -5.75 | 14 | 2 | Y-A |
| **15** | -4.5 | 13.13 | 2 | Y-C |
| **16** | -5.75 | 14 | 1 | N |
| **17** | -5.75 | 8.45 | 0 | N |
| **18** | T | T | T | Y-P |
| **19** | -5.33 | 12.5 | 1.5 | Y-A |
| **20** | -5.375 | 10.5 | 0.5 | N |
| **21** | -5.33 | 12.5 | 1.5 | Y-C |
| **22** | -5.75 | 14 | 1 | Y-C |
| **23** | -4.79 | 11.88 | 1 | Y-A |
| **24** | NR | NR | NR | NR |
| **25** | NR | NR | NR | NR |
| **26** | IC | IC | 2 | Y-C |
| **27** | -3.83 | 14 | 1.5 | Y |
| **28** | -4.0 | 12 | 2 | N |
| **29** | -6.5 | 11.5 | 1.5 | N-RESEARCH |
| **30** | IC | IC | IC | IC |

*NR= no reply, IC= incomplete VIM-TA, T- tractography based VIM-TA, N= No tractography, Y-A= Yes, tractography as an adjunct to anatomical targeting, Y- C = Yes, tractography in conjunction with anatomical targeting; Y-P = Yes, tractography as primary VIM-TA.*

# **Appendix 3:  3D model coronal views**

*3a: 2019 VIM-TAs mapped onto 3D model of thalamic nuclei*


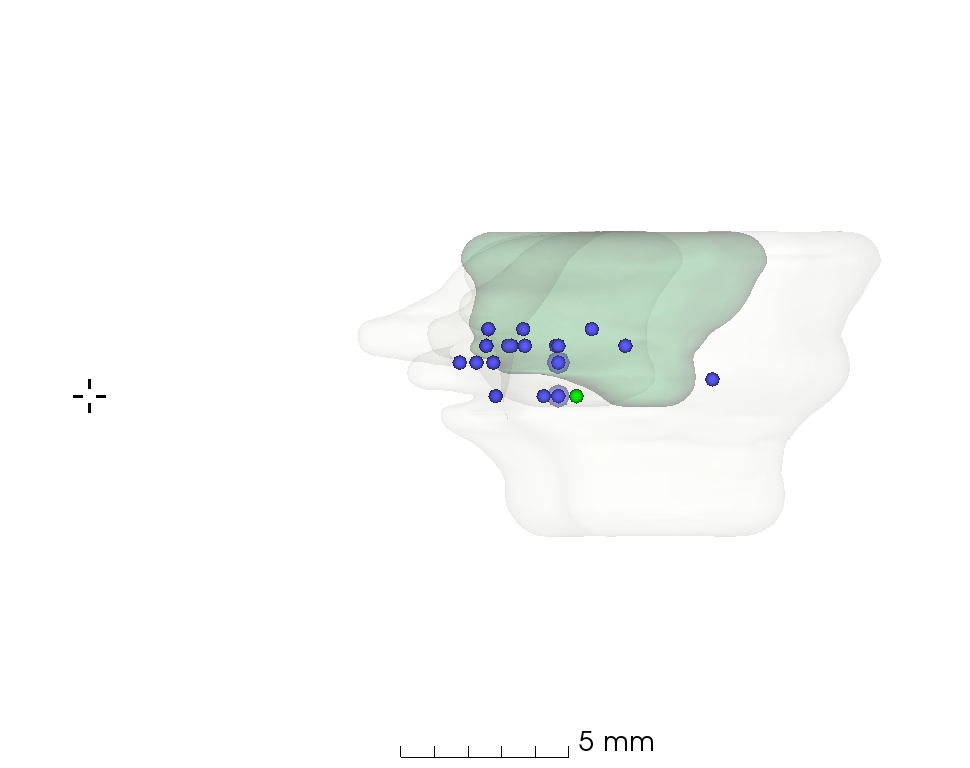


*3b: 2021 VIM-TAs mapped onto 3D model of thalamic nuclei*


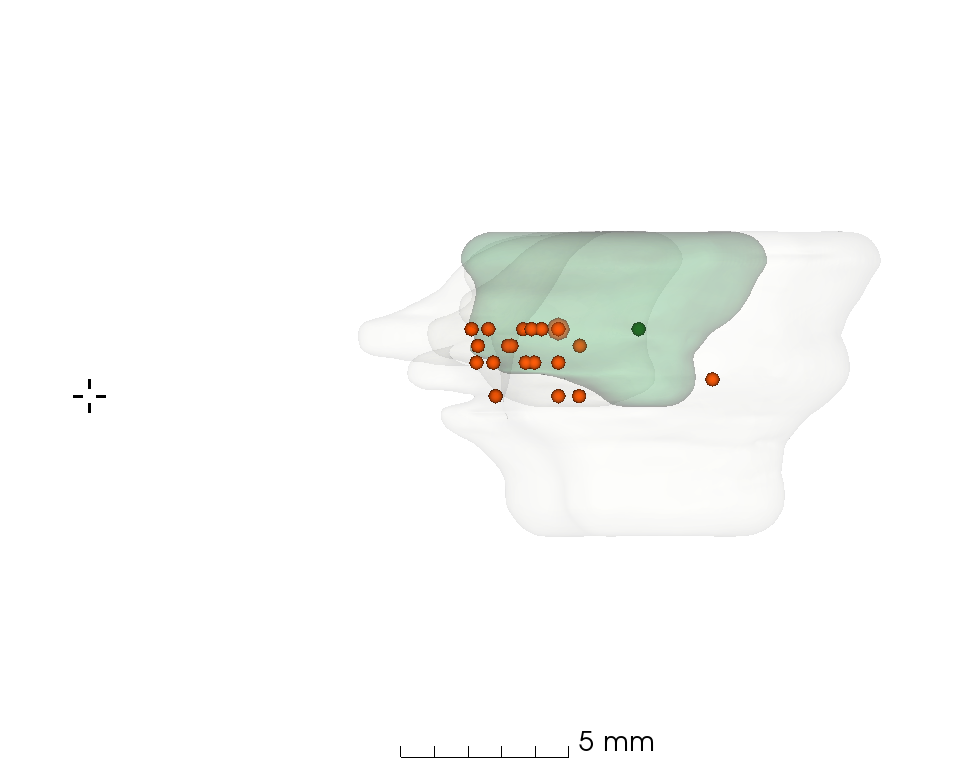


*3c 2019 and 2021: VIM-TAs mapped onto 3D model of thalamic nuclei*


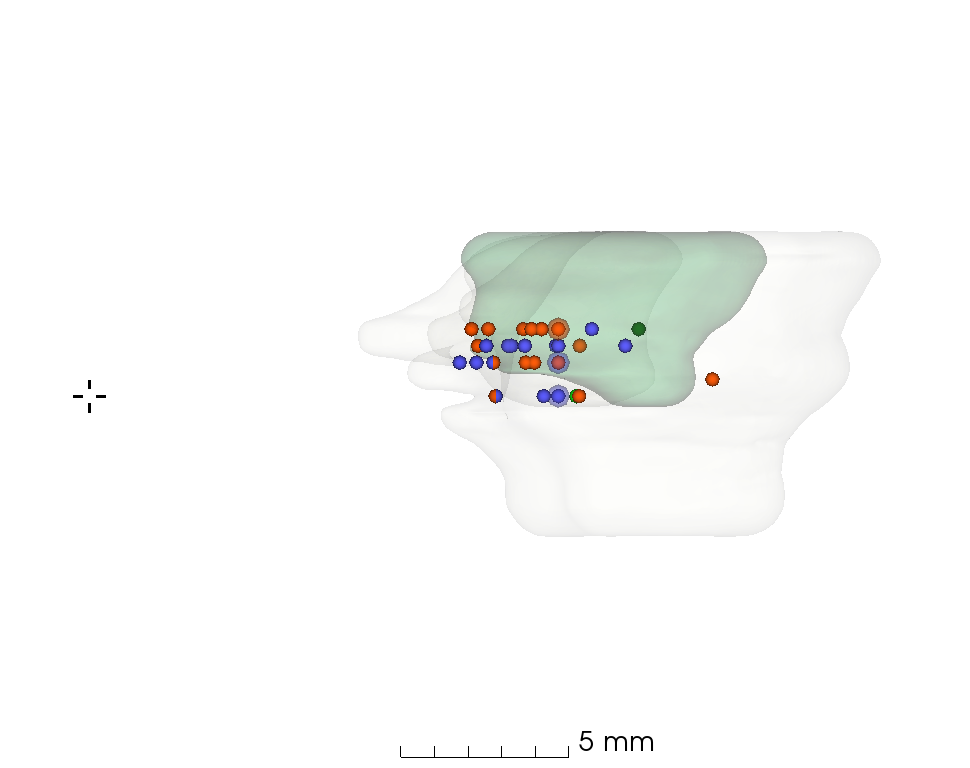


# **Appendix 4:  FUS Centre Rationale for VIM-TA**

| Centre Number | SI coordinate 2021 | QUOTE (selected) |
| --- | --- | --- |
| 1 | 2 mm | *We found tremor relief to be effective when the lesion was centered above the ACPC place, with the bottom margin at or slightly below the plane, and we also found that a lesion at this location was less likely to result in gait imbalance or motor weakness.* |
| 4 | 2 mm | *In the hopes of avoiding ataxia.* |
| 10 | 2 mm | *My rationale for targeting 1.5-2.0 mm above the AC/PC plane was based upon how FUS lesions are created; they expand from the centroid of the target and enlarge peripherally. If I desire to make a lesion @6-8 mm in diameter the lesion would extend down below the AC/PC plane by @ 3-4 mm if targeting at the AC/PC plane so I target above so that it only extends down 1-2 mm. When I first started doing FUS thalamotomies in 2016 I began closer to AC/PC plane and had significant ataxia in my first patient, therefore moved superiorly. This gives less imbalance in patients but preserves the tremor benefit.* |
| 12 | 2 mm | *the choice to move the target 2mm above as an initial coordinate was actually among the earliest adjustments; the main rationale was to have a safety margin for the lower extent of oedema* |
| 13 | 2 mm | *My rationale was that the active electrode for DBS was almost never the bottom electrode, which was typically targeted at 0 Z. Also, GKS was targeted typically to 2-3 mm above AC/PC.* |
| 14 | 2 mm | *safety, being at ventral part of thalamus risks spread of lesion into subthalamic region, lesions spread in dorsoventral direction* |
| 15 | 2 mm | *We always start 2mm superiorly to AC-PC line, because the lesion is rather cylinder or oval shape in coronal than round. After than we adjust the target coordinates based on clinical effect.* |
| 26 | 2 mm | *The more cranial target usually shielded good tremor response and less frequently elicited sensory responses* |
| 28 | 2 mm | *At 2mm we elicit good tremor response whilst maintaining safety. Furthermore due to the size of the lesion we are able to target a second site 1mm below the AC-PC line in the posterior subthalamic area. We find tremor suppression has greater longevity with this double lesion technique* |
| 27 | 1.5 mm **moved to 2mm in 2022* | *Actually we have moved our target up to 2mm sup. this year and we think this place is safer.* |
| 19 | 1.5 mm | *The AC-PC level is generally believed to be the bottom border of Vim, as we can see in stereotactic atlases. Therefore, in case of DBS/rediofrequency coagulation, my target is on the AC-PC level so that the "tip" of the electrode, and stim/lesion comes proximal to the tip. The reason I target  1.5 mm above above AC-PC in case of  MRgFUS is  to avoid the lesion extending below AC-PC. I do not think difference btw 1.5 mm and 2 mm does not matter very much. Actually in many patients, the bottom of Vim is higher than AC-PC level, when Vim is visualized with AI mapping technique.* |
| 21 | 1.5 mm | *For SI, we kept the same location  which is 1.5mm above ac-pc plane. If first try did not get totally tremor control, we move a little towards ac-pc plane.* |
| 5 | 1 mm | *I only target higher for a second side procedure.* |
| 22 | 1 mm | *we tried it only recently, especially for intention tremor with good effects* |
| 23 | 1 mm | *We have discussed the issue of moving upward several times in our group, and (although not stated in my answer) in the second part of 2021 we have started verifying the target at 1 mm Sup, but performing ablation at 1,5 mm Sup to try avoiding complications due to cranio-caudal elongation of the heating target. I must admit we never went up to 2 mm Sup. The neurosurgeon in our group has a Gamma Knife background and has performed some VIM radiosurgical ablations using a totally different approach than the one used with MRgFUS (Guiot's technique)  and this may have conditioned the decision not to always move upwards.* |
| 20 | 0.5 mm | *Our target is effective. So we don't have changed our target* |
| 8 | 0 mm | *We move from 0.5 to 1.0 mm posterior and also from 0.5 to 1.0 mm ventrally (i.e., inferior) from the first target. The exact lenght of movement within this range depends onto specific characteristics of the patient (anatomy, atrophy, head size..), of the treatment (presence of side effects (if we have any hint of limb ataxia by do not move ventral), sonication time in the first target (for instance, if we have delivered long sonications we usually move further to avoid excessing overlap)...), and also imaging characteristics of the lesion in the first target accoirding to intra procedure MRI.  The rationale is based on the fact that the data suggest (Boutet 2018, Pineda-Pardo 2019) that the sweet spot for tremor improvement lays ventral and posterior to the Vim and, therefore, we try to impact onto it. Also, by doing a single target there is more probability of relapse because the lesions might be too small. In our experience (and accordign to the literature) enlarging the lesion in that direction has a good benefit-to-risk balance* |
| 9 | 0 mm **moved to 1mm in 2022* | *we seem to be getting similarly good results starting 1mm above AC-PC, its slightly further form the internal capsule and gives flexibility to create an additional lesion at the AC-PC plane if required. We have looked at tractography images concurrent to surgical planning when we had the tractography software availble in real time, but more for interest as a guide rather than routinely incorporating them into our planning. We are looking at DTI tractography carefully in every patient as research tool to understand mechanisms of outcome and as possible predictor of  short and long term outcome* |
| 17 | 0 mm  **moved from 1.5mm in 2019* | *Before I started to do HiFUS Vim ablations, I targeted the Vim at x=14 (adapted to width of lateral ventricle), y=25% ACPC distance anterior of PC and z=ACPC level.  That is where I placed the lowest contact of the Medtronic 3389 electrode (making the contacts spaced by 2 mm). Mostly, we stimulated  the second lowest contact. When I started to do HiFUS ablations, I had the idea to copy experiences from DBS and performed a DBS-like targeting, including the trajectory starting from around the Kocher’s point. This way, the active contact would be localized about x= 14.5, y=26% anterior PC, z= 2 mm above ACPC.  When we ablated the vim thus, we had a good response of the tremor, but relatively frequent side effects regarding sensitivity. Several patients reported hypesthesia of the tongue or perioral region. Thus, I tried to ablate at ACPC level at the coordinates described above. We had only very rare  hypaesthesias since and also good tremor control. During a visit in* ***XXXX*** *learned, that* ***XXXX*** *is even more aggressive and regularly enlarges his target into the subthalamic region. We started to copy that, but not regularly. As long as we have sufficient tremor control at the first target, we leave it like that. If tremor control is insufficient and the patient shows no side-effects (mostly ataxia or dysmetria), we add a second target 1mm below and 1 mm posterior of the first target for a second ablation. The rationale is to add ablation of fibers of the CTT in addition to the Vim.* |

***XXXX*** *redacted to preserve anonymity*

# **Appendix 5: Detailed Discussion**

***Effect of experience on VIM-TA***

There was a correlation between experience and VIM-TA position in the SI plane. 57.1% of centres who had performed more than 100 treatments targeted at 2mm above the AC-PC line, compared to 33% of centres who had performed less than 50 treatments (Figure 5a). There was an inverse correlation between experience and movement of VIM-TAs; 66.7% of centres that performed less than 50 treatments moved their targeting approach across the study period compared to 28.7% who had performed between 50 -100 procedures. No centres with experience of more than 100 FUS procedure experience moved across the study period. (Figure 5b).

***Effect of Geography on VIM-TA***

Geography appeared to impact VIM-TAs (Fig 6), with a higher proportion of R1 (North American) centres evolving their practice superiorly. In 2019, 20% of R1 and 25% of R2 (European) US centres targeted VIM at 2mm above ICL. In 2021, this increased in R1 to 66.7% but was relatively stable in R2 at 28.6%. Most R4 (East Asia and Australia) centres did not move their VIM-TA superiorly across the study period, although the majority, 66.7%, were already targeting at 1.5mm above ICL in 2019. This changed in 2021 to 50.0% with an additional to 16.7% targeting at 2mm above ICL.

***The rationale for changing practice***

The rationale for changing practice from centres whose VIM-TAs evolved over the study period was varied and are detailed in Table 2 and Appendix 4. Regardless of their 2021 SI co-ordinate, many centres who reported their rationale reported tremor suppression (Category 1) as their main reason for their VIM-TA. 66.7% of those targeting at ICL and 77.8% of those targeting at 2mm above ICL reported “reduce adverse effects or for safety” in their rationale (Category 2). Interestingly 100% of those targeting at ICL reported “to allow second target” (Category 3) in their rationale suggesting many centres do second VIM ablation or a PSA ablation if adequate tremor suppression is not achieved at ICL. Of note, only 11.1% of those targeting at 2mm above ICL, reported a second or subsequent target.
